# Remembering Donald F. Steiner

**DOI:** 10.3389/fendo.2015.00057

**Published:** 2015-04-30

**Authors:** Michael A. Weiss, Shu Jin Chan

**Affiliations:** ^1^Department of Biochemistry, Case Western Reserve University School of Medicine, Cleveland, OH, USA; ^2^Department of Medicine, Case Western Reserve University School of Medicine, Cleveland, OH, USA; ^3^Department of Medicine, University of Chicago School of Medicine, Chicago, IL, USA

**Keywords:** proinsulin, diabetes mellitus, insulin receptor, prohormone processing, hormone biosynthesis

Pioneering endocrinologist and molecular biologist Donald Frederick Steiner, A. N. Pritzker Distinguished Service Professor of Medicine and Biochemistry and Molecular Biology at the University of Chicago (Figure 1A), died on November 11, 2014 at the age of 84. “Very few scientists,” observed Nobel Laureate Joseph L. Goldstein, MD, Chairman of the Department of Molecular Genetics at the University of Texas (UT) Southwestern Medical Center, “can lay claim to original research that has stood the test of time in both the biological and clinical arenas.” Steiner’s contributions meet this dual standard. Dr. Steiner was also renowned for his mentorship of successive generations of leaders in biochemistry and academic medicine.

**Figure 1:**
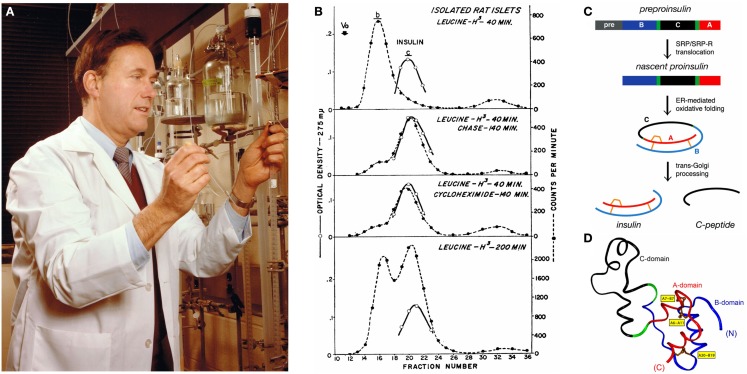
**Discovery of proinsulin**. **(A)** Steiner in his laboratory at the University of Chicago in the mid-1970s. Gel-filtration column chromatography enabled separation of proinsulin, insulin, and C-peptide. **(B)** Chromatograms documenting the transformation of proinsulin to insulin in isolated islets of Langerhans as described in the landmark paper of 1967 (1). *Top panel*, elution pattern of ^3^H-Leu labeled acid-alcohol soluble protein extracted after incubation for 40 min. *Middle two panels*, transfer of radioactivity from peak b (proinsulin) to c (insulin) during subsequent 140 min in presence, respectively, of cycloheximide or 100-fold excess of unlabeled l-leucine. *Bottom panel*, pattern of radioactivity after 200 min incubation without intervention. Optical density (vertical axis at left) pertains to added bovine insulin as control. Chromtography employed G-50 Sephadex. **(C,D)** Biosynthesis of proinsulin. **(C)** Pathway begins with preproinsulin (top): signal peptide (gray), B-domain (blue), dibasic BC junction (green), C-domain (black), dibasic CA junction (green), and A-domain (red). Specific disulfide pairing in the ER yields native proinsulin (middle panels). BC and CA cleavage (mediated by prohormone convertases PC1 and PC2) releases insulin and C-peptide (bottom). **(D)** Solution structure of proinsulin: insulin-like moiety and disordered connecting peptide (black line). A- and B-domains are shown in red and blue, respectively; C-domain contains a nascent α-helical turn near the CA junction. Cystines are labeled in yellow boxes. This figure was obtained from Weiss (2) with permission of the author.

For over five decades, Don Steiner maintained an active research laboratory, produced over 300 peer-reviewed papers, and made important contributions that advanced the fields of diabetes research, protein precursor processing, peptide biology, and hormone evolution and action, a record of productivity that has been matched by few scientists. His discovery of proinsulin in 1967 – a milestone in peptide hormone biology – established proprotein biosynthesis and proteolytic processing as an important field of study (1). Seminal pulse-chase studies of protein synthesis in pancreatic β-cells (Figure 1B) deciphered the biosynthetic pathway of proinsulin (Figure 1C), leading to determination of its structure (Figure 1D).

Following the discovery of insulin in 1921 by Frederick G. Banting and Charles H. Best in the laboratory of John J. R. Macleod (and its further purification in collaboration with James B. Collip), the clinical importance and availability of this hormone made it an attractive target for biochemical studies. This early era of discovery culminated in the complete amino-acid sequence determination of the mature hormone by Frederick Sanger in 1955; these Nobel-recognized studies showed that insulin is composed of two peptide chains (designated A and B) linked by disulfide bonds. However, the primary sequence immediately raised a fundamental problem in biosynthesis: were the two chains synthesized separately and recombined post-translationally, or was a precursor (“proinsulin”) synthesized and proteolytically cleaved to form the mature hormone? This question was put into sharp relief following international efforts in the 1960s to synthesize insulin through chemical methods. In preparations of synthetic insulin (with purity amenable to crystallization) independently obtained in the laboratories of Panayotis G. Katsoyannis (University of Pittsburgh), Niu Jingyi and colleagues (Academia Sinica Institute of Biochemistry, Shanghai), and Helmut Zahn (German Wool Research Institute in Aachen; Deutsches Wollforschungsinstitut), the final synthetic step was remarkable for its inefficiency: combination of the separate A- and B-peptide chains to achieve specific disulfide pairing. Indeed, the structure, stability, and activity of insulin requires formation of three such bridges, two between chains (cystines A7–B7 and A20–B19) and one within the A chain (cysteine A6–A11) as illustrated in Figure 1D (gold boxes).

To solve this problem, Steiner adopted a synergistic approach that exemplified the productive potential of a collaboration between clinical investigation and basic science: a surgical specimen obtained from a patient with a pancreatic β-cell tumor (insulinoma) was studied in concert with an animal model (rat islet extracts), and then frontier biochemical methods (chromatographic resolution of the protein products of pulse–chase assays) were used to identify and characterize proinsulin (illustrated in Figure 1B). “This finding was a bolt from the blue,” remembered Katsoyannis, “as we had been operating under the assumption that the cellular biosynthesis of insulin would mirror its chemical synthesis.” It is a further tribute to Steiner’s insight as a physician-scientist that he immediately recognized the clinical utility of proinsulin and the C-peptide (derived from the peptide segment in the proinsulin molecule that connects its B and A domains), leading to the development (with Arthur H. Rubenstein) of radioimmunoassays for these molecules. The C-peptide assay is still in use to assess residual β-cell function in patients and track its preservation or decline in the natural history of both type 1 and type 2 diabetes mellitus.

In subsequent years, Steiner both continued to characterize proinsulin and expanded his research to other areas which, although insulin-focused, utilized cutting-edge techniques in molecular biology, in part developed in-house. Together with student Susan Terris, Steiner was thus the first to show that insulin clearance in the blood is mediated by its binding to the insulin receptor. Further, the Steiner laboratory was among the first to clone and characterize the convertase enzymes that process pro-proteins. As a physician-scientist, Steiner also helped to identify a new class of monogenic diabetes syndromes due to mutations in the insulin gene, a program of research first led at the protein level by the late Howard S Tager^1^ and enriched by the clinical insight of Rubenstein. Steiner’s interest in monogenic diabetes syndromes also led (in collaboration with colleague Graeme I. Bell) to the characterization of clinical mutations in the insulin receptor gene. Recent studies by the Chicago team of Bell, Steiner, and Louis H. Philipson extended this program to identify a novel class of mutations that perturb the nascent folding of proinsulin, a major molecular mechanism of β-cell dysfunction in permanent neonatal-onset diabetes.

Steiner combined his long-term interest in diabetes-related molecular genetics with foundational studies in evolutionary biology. The sequence, structure, and function of insulin-like proteins provided a model for probing the origins, evolution, and divergence of a gene family. With one of the present authors (Shu Jin Chan), Steiner presented evidence that insulin and related insulin-like growth factors evolved from a common ancestral gene early in chordate evolution. This work anticipated the discovery by several laboratories of an insulin-related superfamily of protein folds in metazoans (including fruit flies and nematodes) whose rich biology continues to be a source of discovery.

Steiner kept in his office a three-dimensional atomic model of the insulin molecule (Figure 2A). Evocative in its artistry, this model was a gift of the late Nobel Laureate Dorothy C. Hodgkin (*inset* at upper right in Figure 2A) whose Oxford laboratory’s pioneering crystallographic analysis of the insulin hexamer came to fruition just after the discovery of proinsulin.^2^ “Don made a very original and generous contribution to our science,” reflected Sir Thomas L. Blundell (Sir William Dunn, Professor of Biochemistry at Cambridge University), at that time a graduate student. “The discovery of proinsulin had a huge influence on the way that the Hodgkin group in the 1960s – Guy Dodson, Eleanor Dodson, [Mamannamana] Vijayan, and me – thought about insulin as we sought to understand the relationship of sequence, structure, folding, and function.” This model depicted insulin as a *monomer* (as extracted from the crystallographic hexamer) and so highlighted the central and still elusive question of how the monomeric hormone binds to and activates the insulin receptor, which is a homodimer (Figure 2B). To address this question, Steiner was a key member of an international team (also involving the present authors) that recently obtained crystal structures of insulin bound to fragments of the insulin receptor (3, 4). “I have very fond memories of my conversations and interactions with him over the past 15 years as we began to tease out the nature of the insulin/receptor interaction,” remembered Colin Ward [founder of the receptor crystallographic group at the Commonwealth Scientific and Industrial Research Organization (CSIRO) in Melbourne, Australia]. “What a true inspiration he was with his enthusiasm for science to the end.”

**Figure 2:**
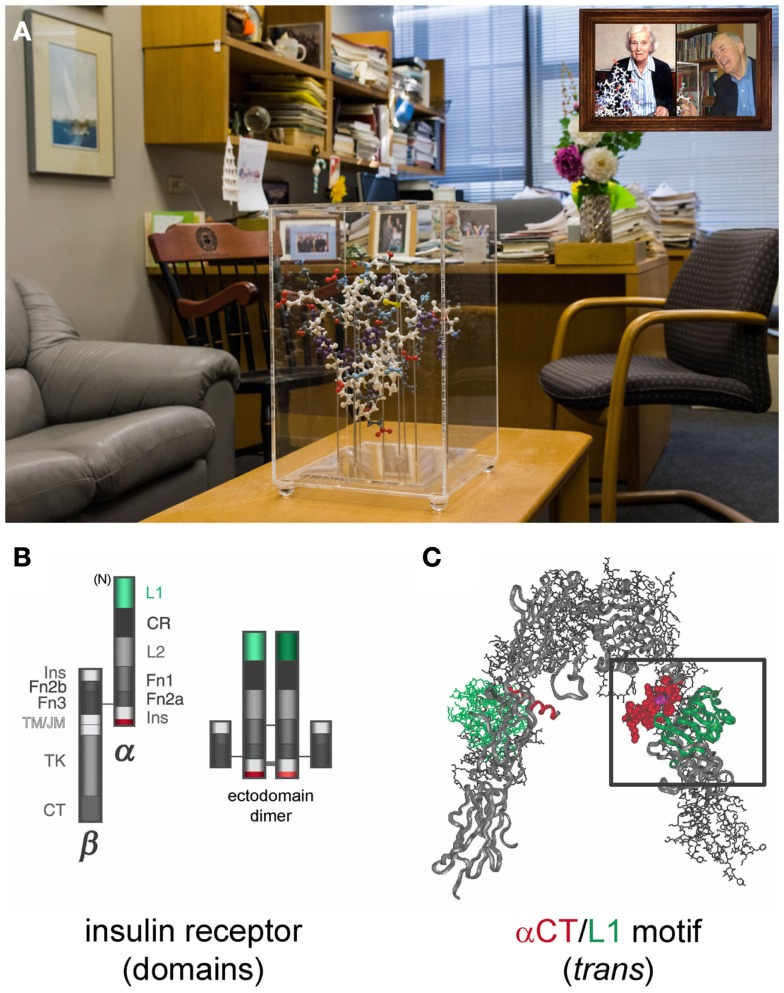
**An international structural team**. **(A)** Steiner’s office at the University of Chicago with Oxford model of insulin in its classical crystallographic conformation (foreground). *Inset at top right*, Hodgkin and Steiner with matching molecular models. **(B,C)** Next structural frontier: the tandem hormone-binding site of the insulin receptor. **(B)** Domain organization of the disulfide-bridged αβ monomer (*left*) and ectodomain (*right*), comprising (αβ_Δ_)_2_ dimer wherein β_Δ_ represents a fragment lacking transmembrane α-helix and intracellular domains. L1 and αCT are highlighted in green and red (*left*), respectively. Domains (gray scale) are otherwise designated cysteine-rich (CR), second Leu-rich repeat domain (L2), type III fibronectin-homology domains (Fn1-3), insert domain (Ins, split between C-terminus of α subunit (bottom) and N-terminus of β subunit (top)), transmembrane and juxtamembrane regions (TM/JM), tyrosine kinase (TK), and C-terminal segment (αCT; 704-FEDYLHNVVFV-715; α-helix bold). Disulfide bridges are shown as horizontal lines. **(C)** Crystal structure of ectodomain. One αβ_Δ_ protomer is shown as a ribbon; the other as sticks. L1 and αCT in each protomer are highlighted in green and red. Electron density of ID-N is incomplete. This figure was adopted with permission from Whittaker (6). Coordinates of the ectodomain of the insulin receptor were obtained from Protein Databank entry 3LOH as described (7).

Critical to the recent crystallographic efforts was Steiner’s prescient identification in 1994 of a novel hormone-binding element within the insert domain (labeled “Ins” in Figure 2B) at the extreme C-terminus of the receptor α subunit (highlighted in red). This conserved element, designated αCT, is shared by the Type 1 IGF receptor but not, to our knowledge, other classes of receptor tyrosine kinases. The tandem motif of αCT (red α-helix in Figure 2C) and the N-terminal leucine-rich domain of the receptor α subunit (L1 domain; green in Figure 2C) defines the primary hormone-binding site of the ectodomain (boxed). The identification of αCT by residue-specific hormone-receptor photo-crosslinking by the collaborating laboratories of Steiner and Katsoyannis represented a *tour de force* of synthetic peptide chemistry and analytical biochemistry (5). “Methods for the total chemical synthesis of insulin,” recalled Katsoyannis, “enabled preparation of unique research reagents to probe photo-contacts by individual residues. This extended photo-chemistry from small peptides to a globular hormone^3^.”

To achieve these results over five decades, Steiner strove to create a laboratory that was structured but also allowed scientific freedom for the training of physician-scientists and basic investigators. “The atmosphere in his laboratory was pleasant, but the planning, execution, and evaluation of experiments was rigorous and meticulous,” remembered former fellows Rubenstein (Dean *Emeritus* of the University of Pennsylvania School of Medicine) and Kenneth S Polonsky (Dean of the Pritzker School of Medicine at the University of Chicago). “He spent hours worrying about unexpected results and repeated experiments numerous times before being satisfied. We all felt part of his team and when new discoveries were made, we all shared in the joy of the science and celebrated together.” During the initial characterization of proinsulin, however, Steiner felt constrained to limit projects that the group could pursue. “It was one of the hardest things that I ever had to do,” he said to one of the present authors (Shu Jin Chan), “since I’ve always felt that students should be free to explore their creative ideas.” It was this focus at a seminal time in the history of molecular endocrinology that enabled the paradigm of prohormone processing to be established.

In recognition of his scientific achievements, Steiner received numerous awards and prizes, including the Gairdner Award (1971), Diaz-Cristobal Award (1973), Banting Medal (1976), Borden Medal (1980), Wolf Foundation Prize (1985), Fred C. Koch Award (1990), and Manpei Suzuki Prize (2009). He also received honorary degrees from the University of Umea (Sweden), University of Illinois (Chicago), Rheinisch-Westfalische Technische Hochschule (Germany), University of Uppsala (Sweden), Mount Sinai School of Medicine (New York), and University of Copenhagen (Denmark). Steiner was for many years a member of the Editorial Board of the *Journal of Biological Chemistry* and a founding Associate Editor of this journal.

Don Steiner was born in 1930 in Lima, Ohio and in 1952 received a BS in Chemistry and Zoology from the University of Cincinnati. In 1956, he obtained an MS in Biochemistry and MD from the University of Chicago. Following an internship at King County Hospital (Seattle) and residency and post-doctoral training at the University of Washington, Steiner joined the Department of Biochemistry at Chicago in 1960. Promoted to professor in 1968 and department chair in 1973, he was a Senior Investigator in the Howard Hughes Medical Institute (HHMI) from 1985 to 2006. Steiner was also Director of the University of Chicago Diabetes-Endocrinology Center (1974–1978) and for 40 years served as the leader or coleader of the University’s NIH-designated Diabetes Research and Training Center (DRTC). Steiner was a member of the American Academy of Arts and Sciences (elected in 1972), the National Academy of Sciences (1973), and the American Philosophical Society (2004).

Steiner’s pioneering dissection of the molecular mechanisms of insulin biosynthesis provided broad insight into cell biology and protein evolution. “Fifty years ago,” concluded Goldstein, “Don Steiner discovered that insulin in the blood is derived from a larger precursor molecule. This exceptionally original work profoundly influenced the thinking of biologists, leading to the concept of proteolytic processing as a mechanism for generating regulatory peptides.” Yet, his legacy extends beyond even these landmark investigations. “Don Steiner had a broad and profound impact as a result not only of his groundbreaking scientific discoveries,” remembers Polonsky, “but also because of his generosity in helping colleagues, collaborators, and particularly students and other trainees to raise the level of their science.” We shall miss him both as a brilliant scientist and colleague of profound humanity.

## Conflict of Interest Statement

The authors declare that the research was conducted in the absence of any commercial or financial relationships that could be construed as a potential conflict of interest.

## Footnotes

^1^Tager was a post-doctoral fellow with Steiner from 1971 to 1973. He returned to the University of Chicago in 1974 as an Assistant Professor of Biochemistry and Molecular Biology and rapidly rose through the ranks. At the time of his death in 1994, Tager was Louis Block Professor and Department Chair.

^2^Eleanor Dodson (Professor of Chemistry at the University of York, York, UK) recalls that the model was a gift from Hodgkin. In the years after the discovery of proinsulin and determination of the insulin crystal structure, Steiner, Hodgkin, and Dorothy’s former students (Guy and Eleanor Dodson at York; and John F. Cutfield and Sue M. Cutfield, University of Otago, Dunedin, NZ) regularly visited each other’s laboratories. “Don was a very good friend as well as a great scientist,” Eleanor Dodson shared. “We had some very happy times with him.” Through the Dodsons, this network of interactions was extended to Martin Karplus (Theodore William Richards, Professor of Chemistry *Emeritus* at Harvard University and recipient of the 2013 Nobel Prize in Chemistry) who encouraged studies of insulin by NMR spectroscopy and molecular-dynamics simulations.

^3^Katsoyannis emphasized the complementary contributions of several laboratories to the development of synthetic (or semi-synthetic) insulin photo-chemistry, including D. Brandenburg, S. E. Shoelson, and the late C. C. Yip.
